# Interactions affect hyphal growth and enzyme profiles in combinations of coniferous wood-decaying fungi of Agaricomycetes

**DOI:** 10.1371/journal.pone.0185171

**Published:** 2017-09-27

**Authors:** Tuulia Mali, Jaana Kuuskeri, Firoz Shah, Taina Kristina Lundell

**Affiliations:** Microbiology and Biotechnology, Department of Food and Environmental Sciences, Viikki Campus, University of Helsinki, Helsinki, Finland; USDA Forest Service, UNITED STATES

## Abstract

*Fomitopsis pinicola* is a species of Polyporales frequently encountered in Nordic temperate and boreal forests. In nature, the fungus causes destructive brown rot in wood, colonizing tree trunks often occupied by other Basidiomycota species. We mimicked these species-species interactions by introducing *F*. *pinicola* to five white rot species, all common saprotrophs of Norway spruce. Hyphal interactions and mycelial growth in various combinations were recorded, while activities of lignocellulose-acting CAZymes and oxidoreductases were followed in co-cultures on two different carbon-source media. Of the species, *Phlebia radiata* and *Trichaptum abietinum* were the strongest producers of lignin-modifying oxidoreductases (laccase, manganese peroxidase) when evaluated alone, as well as in co-cultures, on the two different growth media (low-nitrogen liquid medium containing ground coniferous wood, and malt extract broth). *F*. *pinicola* was an outstanding producer of oxalic acid (up to 61 mM), whereas presence of *P*. *radiata* prevented acidification of the growth environment in the liquid malt-extract cultures. When enzyme profiles of the species combinations were clustered, time-dependent changes were observed on wood-supplemented medium during the eight weeks of growth. End-point acidity and production of mycelium, oxalic acid and oxidoreductase activities, in turn clustered the fungal combinations into three distinct functional groups, determined by the presence of *F*. *pinicola* and *P*. *radiata*, by principal component analysis. Our findings indicate that combinations of wood-decay fungi have dramatic dynamic effects on the production of lignocellulose-active enzymes, which may lead to divergent degradative processes of dead wood and forest litter.

## Introduction

Fungal interactions are dynamic processes in nature comprising not only pathogen-host and symbiotic relationships, especially with plants, but also microbe-fungal and fungal-fungal interspecies level activities in diverse habitats. Of these habitats, one of the most demanding environments is dead wood [1]. In regard to the degradative saprotrophic (saprobic) processes, the fungal community in decaying wood and woody debris may be under dynamic changes in the forest ecosystems [2–5]. Interactions between fungal species (interspecies activities and responses) affect—either positively or negatively—the community composition and wood colonization strategies [6–7]. Thus, wood-decay fungi may confront each other in their habitats with several strategies that are either mutualistic, neutral, or combative (antagonistic) hyphal interactions [6, 8].

Competition between fungal hyphae of different species or intraspecies isolates arises from competition of environmental resources, which is demanded by whether the resource territory is already occupied or not [6]. The local abiotic and biotic factors are the driving forces for fungal interactions, and the initial colonizers may create a priority effect on the success of later occupants [4–5, 9]. Furthermore, availability of readily degradable and utilizable organic compounds, such as plant litter and wood in the forest ecosystems, affect hyphal colonization [8]. To gain nutritional carbon from solid wood—the main lignocellulose material in nature—saprotrophic fungi secrete a mixture of metabolites and enzymes to break and modify the cellulose, hemicellulose, pectin, and lignin biopolymers of the composite wood cell wall structures [10–12].

In dead wood, the wood-decaying fungi belonging to Basidiomycota class Agaricomycetes are able to colonize and even decompose compact wood and lignified plant cell walls by production of multiple arrays of carbohydrate active enzymes (CAZymes) and auxiliary oxidoreductases [11–12]. Expression and secretion of a wide array of these enzymes builds up the white rot fungal decay system [13–15]. Another type of efficient degradation of wood is the fungal brown rot decay, which is deficient of the key white rot signature enzymes such as class-II peroxidases [10–11, 16] but is distinguished by a strong oxidative non-enzymatic attack leading to destruction of wood cellulose and polysaccharides [17–18].

Lignin is the most recalcitrant of the plant biopolymers, and it is attacked and modified by oxidoreductase enzymes secreted by the white rot fungi [11–12, 14, 19]. Evolution of lignin-modifying ability was initiated (around -290 Ma) when the first class-II peroxidase encoding genes appeared in the ancestors of Agaricomycetes [11]. The main wood polysaccharides—cellulose and hemicelluloses–in turn are decomposed to oligo- and monosaccharides by fungal secreted CAZymes including e.g. endoglucanase, xylanase, β-glucosidase, cellobiohydrolase and lytic polysaccharide monooxygenase activities [20].

Contrary to the white rot Agaricomycetes, it is assumed that brown rot fungi primarily decompose the wood polysaccharides—cellulose and hemicelluloses–non-enzymatically by generating reactive oxygen species *via* Fenton chemistry [17–18,21–22], while expression and functions of cellulose and hemicellulose degrading CAZymes has been studied with a few model species of brown rot fungi [23]. Fenton reactions initiate wood decay by opening up the xylem cell wall structure, which is followed by CAZyme expression [23]. According to the accumulating fungal genomic information and phylogenomics, previous categorization of wood-inhabiting fungi to either white or brown rot species is, however, now deforming into several, more diverse fungal mechanisms and observable characteristics [12–13,15, 24]

Fungal communities in decaying wood have been investigated by molecular approaches (DNA sequence-amplicon based fungal diversity studies) pinpointing combinations and succession of a few white rot and brown rot species, together with ectomycorrhizal and Ascomycota species, in dead coniferous and deciduous tree species [5, 25–28]. However, only a few studies were targeted on enzyme activities in decaying wood [29], or were conducted under controlled conditions including fungal species-species interactions [7, 30].

For these reasons, and taking into account the recent fungal community studies, we designed specific and ecologically justified wood-decay fungal species interaction experiments under laboratory conditions, applying both solid media (agar plates) for detection of hyphal interactions, and coniferous wood-containing semi-solid cultures to follow the effect of different interactions on the production of wood carbohydrate-active and lignin-modifying enzymes. The main purpose was to investigate, how the presence of the brown rot species *Fomitopsis pinicola* (order Polyporales) influenced hyphal growth and secretion of extracellular wood-decay enzymes by white rot species of Polyporales (*Phlebia radiata*, *Trichaptum abietinum*, *Junghuhnia luteoalba*) and Hymenochaetales (*Porodaedalea laricis*, former *Phellinus chrysoloma*; *Phellinus ferrugineovelutinus*, former *Phellinus ferrugineofuscus*).

The selected Agaricomycetes species are well known for their ability to colonize and decompose dead wood, especially Norway spruce, in boreal forests [26, 31–34] (Table 1). Our research hypothesis was that the brown rot fungus would have a strong, either inhibiting or promoting effect, on hyphal growth and production of wood-decay enzymes by the white rot species, which would be observable in different species combinations and co-cultivations.

**Table 1:** Fungal species systematics, wood-decay type and decay stages.

Fungal species, abbreviation and classification	Isolatecode^1^	ITS sequence accession^2^	Wood-decay type^3^	Wood-decay stage^4^	Ref.
*Fomitopsis pinicola* (Sw.) P.Karst. (1881) (Fp) Fomitopsidaceae, Polyporales	1181	LT844580	BR	Early	[35]
*Trichaptum abietinum* (Dicks.) Ryvarden (1972) (Ta) Polyporaceae, Polyporales	0110	LT844582	WR	Early	[25]
*Phlebia radiata* Fr. (1821) (Pr) Meruliaceae, Polyporales	0043	LT844581	WR	Early, secondary	[1,36]
*Junghuhnia luteoalba* (P.Karst.) Ryvarden (1972) (Jl) Meruliaceae, Polyporales	1472	LT844583	WR	Middle	[37]
*Porodaedalea laricis* (Jacz. ex Pilát) Niemelä (2005) (*Phellinus chrysoloma*) (Pc) Hymenochaetaceae, Hymenochaetales	0768	LT844584	WR	Early	[38]
*Phellinus ferrugineovelutinus* (Henn.) Ryvarden (1972) (*Phellinus ferrugineofuscus*) (Pf) Hymenochaetaceae, Hymenochaetales	0945	LT844585	WR	Middle	[35]

^1^HAMBI-FBCC, HAMBI Fungal Biotechnology Culture Collection

^2^This study, see text in S1 File

^3^BR, brown rot; WR, white rot

^4^according to formation of fruiting bodies (basidiocarps) on decaying wood, as is described in the references.

## Materials and methods

### Fungal isolates and culture conditions

Basidiomycota isolates of the class Agaricomycetes, order Polyporales, species *Fomitopsis pinicola*, *Phlebia radiata*, *Trichaptum abietinum*, and *Junghuhnia luteoalba*, and two species of the order Hymenochaetales from the genera *Phellinus* and *Porodaedalea* (Table 1), were obtained from the University of Helsinki Fungal Biotechnology Culture Collection (HAMBI/FBCC). The fungal strains were originally isolated from basidiocarps growing on decaying wood in boreal forest sites in Finland and identified morphologically. Their identity to species level was confirmed by phylogenetic analysis of the ribosomal DNA ITS region (ITS1+5.8S+ITS2 as the amplified region) (Fig A in S1 File). One isolate of each species was selected for the co-culture experiments according to even and uniform hyphal growth on 2% malt extract agar (MEA) (2% w/V malt extract, Biokar, France and 2% w/V agar, Yliopiston Apteekki), pH 5.5±0.05, at 25°C, in the dark. The fungal isolates were pre-cultivated on MEA in 9 cm diameter petri dishes at 25°C, in the dark for one week before initiation of the co-cultivations.

### Fungal co-cultures and hyphal growth on agar media

For inspection of mycelial interactions and measurement of hyphal growth rates, cultivations on 2% MEA and ABTS agar plates were initiated for single-species, two-species combinations, and for three-species co-cultures (Table 2). All cultivations were performed in three biological replicates (agar plates were inoculated and analysed for each combination and single-species culture). ABTS agar medium contained 5.6 mM glucose, 15 mM KH_2_PO_4_, 2 mM MgSO_4_ • 7 H_2_O, 0.9 mM CaCl_2_ • 2 H_2_O, 2.7 mM ammonium tartrate, 15 mM succinic acid, 0.02% (w/V) yeast extract (LabM), 2.5% (w/V) agar (Yliopiston Apteekki), and 0.025% (w/V) ABTS (2,2'-azino-bis(3-ethylbenzothiazoline-6-sulphonic acid, Sigma-Aldrich) [39]. MEA and ABTS agar plate cultivations were started by transferring a mycelial 5 mm x 5 mm agar plug from pre-cultivated MEA plates. Mycelial plugs were placed on the agar surface systematically and in even distribution from the center of the plate, also in the co-cultures (Fig A in S2 File). Extension of the mycelial front (hyphal growth) for each inoculant was recorded 1–5 times a week, depending on the rate of complete colonization of the agar plate, from three equal segments of the growing mycelium, for a maximum period of eight weeks. Mycelial front extension was measured for each species in the cultivations from the three replicate plates, and the results are presented in (mm d^-1^) as mean values with standard deviation.

**Table 2:** Fungal combinations on agar media, in liquid medium and semi-solid coniferous wood co-cultures. Species abbreviations are depicted in Table 1.

One species	Two species	Three species
Fp Pr Ta Pc Jl Pf	FpPr FpTa FpPc FpJl FpPf	FpPrTa, FpPrPc, FpPrJl, FpPrPf, FpTaPc, FpTaJl, FpTaPf, FpPcJl, FpPcPf, FpJlPf

### Fungal co-cultures in liquid media and sampling

In order to follow the effect of fungal interactions on enzyme production and activities, wood-supplemented LN-AS (wood-LNAS medium) and ME broth medium cultivations were conducted in three parallel 100 ml cultures in 250 ml Erlenmeyer flasks, following the same fungal combination scheme (Table 2). The 2% (w/V) malt extract (Biokar) liquid medium (ME), pH 5.5, was prepared in Milli-Q water. The LN-AS (low nitrogen-asparagine succinate buffered liquid medium, pH 4.5) [40–41] mineral medium was supplemented with 1 g ± 0.01 g (dry weight) of a mixture of coniferous wood (Norway spruce and Scots pine) fine filings (sieved to less than 5 mm particle size) as the carbon source prior to autoclaving (121°C, 15 min, 1 atm). Each flask was inoculated with two 5 mm x 5 mm sized mycelial plug of each fungal isolate pre-cultivated on MEA plates for one week. The stationary submerged flask cultures were incubated for eight weeks at 25°C in the dark. Samples (1–2 ml) were taken from each flask once a week, and stored at -20°C. Samples were quickly thawed and centrifuged (13 000 g, +4°C, 2 min) before measuring the enzyme activities.

Production of mycelial mass in the ME broth cultures was determined after eight weeks of cultivation using fiberglass filters (Whatman GFC) and overnight drying at 140°C. Mycelial dry weight was obtained by subtracting the dry weight of the filter from the total dry weight. Acidity (pH value) of liquid media cultures was measured at the end of the cultivation. Mean average pH values with standard deviation of three replicate cultures are reported.

### Enzyme activity and oxalic acid production measurements

Activities of selected CAZy [42] glycoside hydrolases and auxiliary oxidoreductases were measured from three parallel flask cultures (three biological replicates) of each co-culture species combination and from single-species cultivations. Xylanase, β-glucosidase, laccase, and manganese peroxidase (MnP) activities were determined according to the 96 well plate methods previously developed [41, 43] using Tecan Infinite M200 plate reader spectrophotometer connected to Magellan analytical software (version 7.1 SP1). Xylan from beech wood (1% w/V, Sigma) and 1 mM 4-nitrophenyl β-D-glucopyranoside were used as substrates in the xylanase and β-glucosidase assays, respectively. For xylanase activity assay, the calculated theoretical detection limit was 1.67 μkat/L. For laccase activity measurements, ABTS (31 mM) in 50 mM sodium malonate buffer (pH 4.5) was used as substrate, and formation of the green radical product was followed at 420 nm [44]. MnP activity was kinetically determined in 50 mM sodium malonate buffer (pH 4.5) at 270 nm as oxidation of Mn^2+^ to Mn^3+^ malonate chelates [40–41]. Enzyme activities are reported as mean values (with standard deviation) of the three replica culture samples. For each sample in all enzyme activity assays, three technical replica reactions were measured. Exceptional reactions and values were abandoned according to standard deviation of the mean value. Each 96-well plate contained (i) either positive reference enzyme samples (in three parallel reactions) in the laccase and MnP assays, or (ii) reaction product reference samples, either 4-nitrophenol or xylose, in four different concentrations (each in three parallel reactions) in the β-glucosidase and xylanase assays, respectively. Thus, performance of each individual 96-well plate and data recording were confirmed.

Fungal produced oxalic acid was identified and quantified at the end-point of ME broth cultures applying a UHPLC method optimized for analysis of fungal-produced organic acids from liquid media cultures [45]. However, it was impossible to quantify oxalic acid from the wood-LNAS medium cultures by this method due to interference of the medium components in the chromatographic separation.

### Statistical analyses

In order to observe potential correlation of fungal species combination to production of enzyme activities in the wood-LNAS cultures during the eight weeks’ period of cultivation, and to investigate correlation of cultivation end-point data (in the ME broth cultures), principal component analyses (PCA) were conducted using ggplot2 [46], ggbiplot (GitHub, Inc. 2016), ggrepel (GitHub, Inc. 2016), devtools [47], cluster [48], scales [47] and car [49] packages in the R environment, software 3.2.5 [50]. Evolving clustering of species combinations at each time point (wood-LNAS cultures) and at the end-point (ME broth cultures) was observed by performing a k-means clustering with elbow method, and optimal number of clusters was applied in the PCA calculation analyses [48] using the cluster package [51].

The effect of Pr or Ta in regard to production of β-glucosidase activity in the wood-LNAS cultures (time points 4 to 8), were determined using one-way repeated measurements ANOVA together with Tukey *post hoc* tests by using the IBM SPSS Statistics V22.0 package. The normal distribution of the data set at each time point were tested with Shapiro-Wilk test and sphericity were tested with Mauchly’s Sphericity test. Significance of each fungal species as well as the number of species present in the co-cultures, in regard to production of the highest enzyme activities was tested by UNIANOVA and applying Tukey *post hoc* tests. Significance (*P* value ≤0.05) of level of confidence over 95% is indicated for the results.

## Results

### Hyphal growth and mycelial interactions

**Fig 1:**
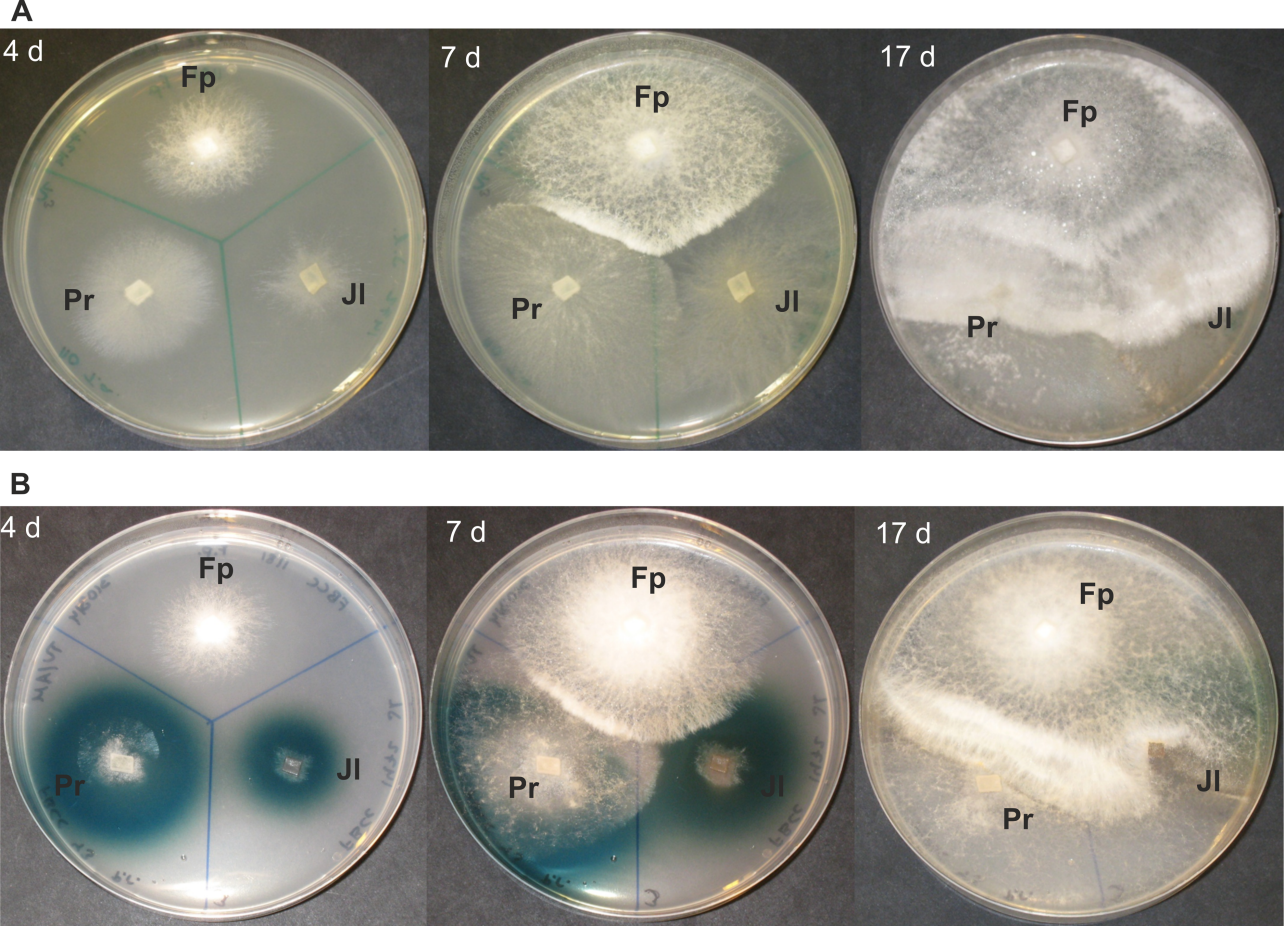
Fungal mycelial interactions recorded after 4, 7 and 17 days of growth on agar media. Co-cultures of three fungal species, *F*. *pinicola* (Fp), *P*. *radiata* (Pr), and *J*. *luteoalba* (Jl) on (A) malt extract agar, and (B) ABTS agar.

In the single-species cultures, *P*. *radiata* was the most prominent in mycelial growth pattern (8.8 mm d^-1^, hyphal front extension) on the MEA reference medium (Table 3). The Polyporales species *T*. *abietinum* and *F*. *pinicola* were also fast in their hyphal extension rates, whereas the two Hymenochaetales isolates (*Porodaedalea laricis*, *Phellinus ferrugineovelutinus*) demonstrated the slowest hyphal extension patterns. In all of the fungal combinations in co-cultures on MEA plates, *F*. *pinicola* demonstrated rapid hyphal growth, competing over the white rot species within 17–41 days depending on the fungal combinations (Fig 1A and 1B; Fig H in S2 File). In most cases on agar media, *F*. *pinicola* hyphae colonized the plate fast extending over the mycelia of the white rot species within 21–30 days forming a white-colored mycelium with dense hyphal fronts upon confrontation (Fig 1A and 1B), suggesting for more of a combative than antagonistic reaction. Of the white rot species studied, according to observations on hyphal growth pattern on agar media, *T*. *abietinum* was the most competitive and *P*. *ferrugineovelutinus* the least antagonistic species against *F*. *pinicola*.

**Table 3:** Hyphal growth rate of fungal isolates on malt extract and ABTS agar media.

Fungal isolate	Malt extract agar (mm/day)	ABTS agar (mm/day)
*F*. *pinicola* 1181 (Fp)	5.5	5.1
*T*. *abietinum* 0110 (Ta)	5.8	2.1
*P*. *radiata* 0043 (Pr)	8.8	4.8
*J*. *luteoalba* 1472 (Jl)	4.5	1.7
*P*. *laricis* 0768 (Pc)	1.7	1.0
*P*. *ferrugineovelutinus* 0945 (Pf)	3.0	1.5

*F*. *pinicola* was the fastest in hyphal extension on the agar media showing similar growth rates on ABTS agar (5.1 mm d^-1^) and on MEA (Table 3). However, only the white rot species produced a diffusible, green-coloured reaction product zone around their mycelia, being indicative of secretion of oxidoreductases–mainly laccase–into the agar medium (Fig 1B). Contrary to this, *F*. *pinicola* mycelium demonstrated no such ABTS-oxidative reactions. Instead, presence of *F*. *pinicola* was noticed to slightly prevent formation of the green zones and ABTS oxidation by the white rot species—except with *J*. *luteoalba*—in relation to the extending hyphae at the later stages of growth (Fig 1B). Otherwise, hyphal interactions between *F*. *pinicola* and white rot species followed similar patterns in the co-culture combinations on both agar media (MEA and ABTS) studied (Fig 1A and 1B).

### Enzyme activities: Laccase

**Fig 2:**
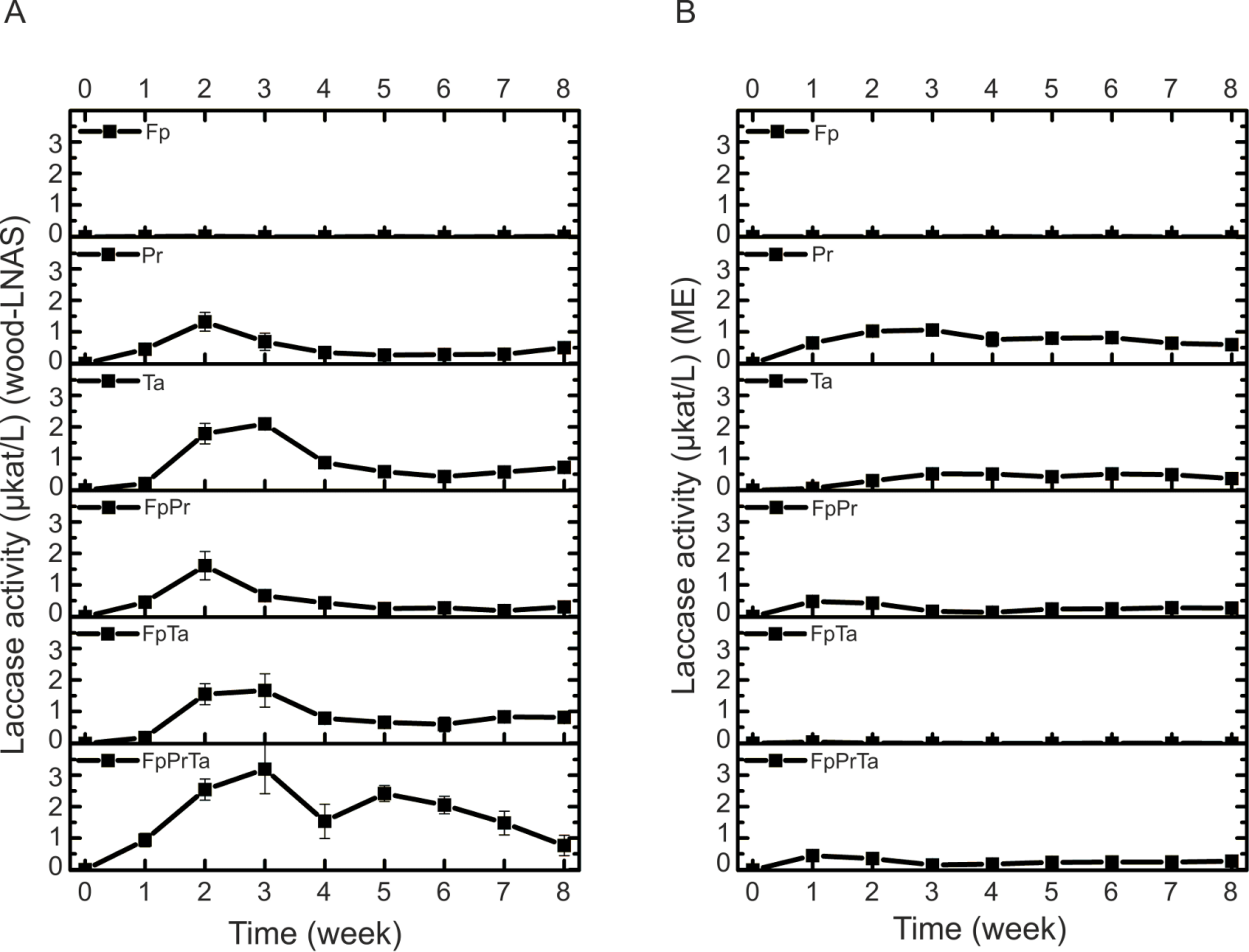
**Effect of *F*. *pinicola* Fp on laccase activities produced in single-species and co-cultures of *P*. *radiata* Pr and *Trichaptum abietinum* Ta during eight weeks of cultivation on (A) semi-solid wood-supplemented LNAS, and (B) malt extract liquid medium.** Mean average values (n = 3, three parallel cultures) with standard deviation are presented at each time point. Fungal abbreviations and combinations, see Table 1 and Table 2.

Since all white rot species caused the formation of green-colour on ABTS-agar indicating expression of laccase activity, it was expected that laccase would readily be generated by the species on the semi-solid coniferous wood-LNAS and more rich liquid ME broth media as well. In wood-LNAS cultures, however, variant activities of laccase–using ABTS as substrate in laccase enzyme assays–were produced by the white rot fungi in co-cultures than in their single-species cultures. The highest activities of laccase (up to 2 and 3 μkat L^-1^) were detected with *P*. *radiata* (Pr) and *T*. *abietinum* (Ta), and in their co-culture combinations with *F*. *pinicola* (Fp), on both wood-LNAS and ME broth media (Fig 2A and 2B). On the contrary, no laccase activity was observed in single-species cultures of Fp on any of the growth media and substrates tested (Fig 2A and 2B; Fig C and Fig D in S2 File).

On wood-LNAS medium, laccase activities produced by Pr and Ta were unaffected by *F*. *pinicola* in the two-species co-cultures (Fig 2A). Similar pattern was observed on wood-LNAS medium in the other three-species combinations of Fp with either Pa or Ta present (Table B and Fig C in S2 File). In the three-species combination of FpPrTa (Table 1 and Table 2) on wood-LNAS medium, however, a very different pattern of laccase activity was observable during the eight weeks’ cultivation period showing activity peaks on week 3 and again on week 5 (Fig 2A), as an early (during weeks 1–4) and a late (during weeks 4–8) phase of combinatory laccase activity. On the more rich (in carbohydrate content) malt extract ME broth, however, the presence of *F*. *pinicola* caused a decline in laccase activities produced by Pr and Ta, which was also observed in their three-species combination cultures (Fig 2B). In contrast to Pr and Ta, only slight laccase activities were noted upon Fp interactions with Pc, Pf and Jl on wood-LNAS medium (Fig D in S2 File), and no laccase activity was detectable in their three-species co-culture combinations (FpPcJl, FpPcPf, FpJlPf; Table B and Fig D in S2 File).

### Enzyme activities: Manganese peroxidase

**Fig 3:**
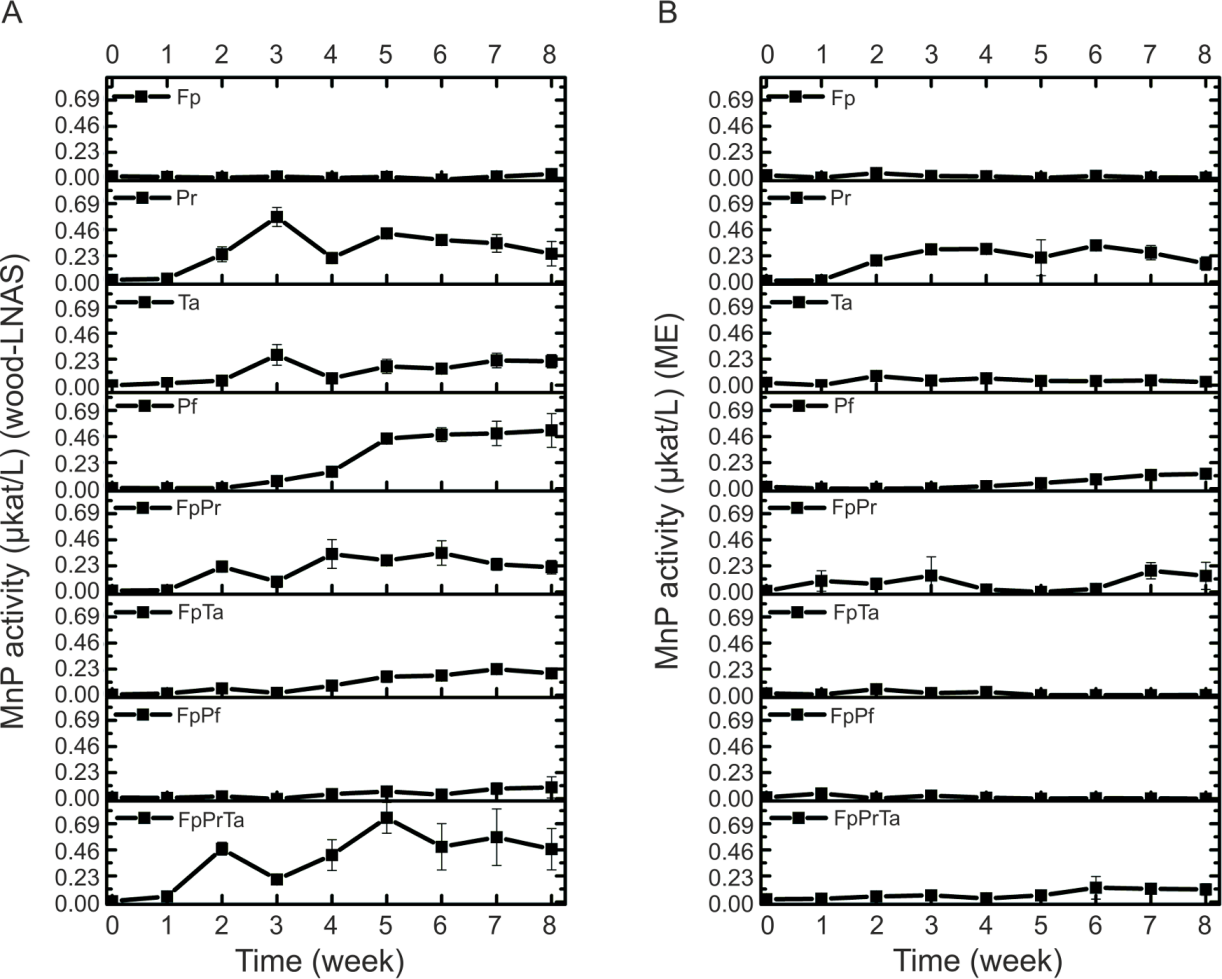
**Effect of *F*. *pinicola* Fp on manganese peroxidase (MnP) activities produced by Ta, Pr and Pf, in single-species and co-cultures during eight weeks on (A) semi-solid wood-supplemented LNAS, and (B) malt extract medium.** Mean average value (n = 3, three parallel cultures) with standard deviation is presented at each time point. Fungal abbreviations and combinations, see Table 1 and Table 2.

Activity of MnP–assayed as oxidation of Mn^2+^ ions to chelated Mn^3+^ complexes–was detectable in all of the white rot single-species cultures on wood-LNAS medium (Fig 3A; Fig E in S2 File), but only in Pr and Pf single-species cultures cultivated on ME broth (Fig 3B; Fig F in S2 File). As expected, no MnP activity was detected in any of the Fp single-cultures, either on wood-LNAS or on ME broth. MnP activities were the most substantial in cultures of Pr and Pf on both liquid media applied (Fig 3A and 3B; Table B in S2 File). Two-phase production of MnP activity was observed in single-species cultures of Pr on both media, but with Fp apparently slightly repressing MnP production by Pr in their two-species co-cultures (FpPr; Fig 3A). Noticeable is the gradual slow increase in MnP production demonstrated by *P*. *ferrugineovelutinus* (Pf) over eight weeks of cultivation, which was observed on both media studied (Fig 3A and 3B). However, no such slow increase in MnP production was observed in the two-species co-culture with *F*. *pinicola* (combination FpPf).

In case of the white rot species Ta, Jl and Pc, MnP activities were observed when the fungi were cultivated on wood substrate (semi-solid ground wood-LNAS medium), with very low or only occasional activity detected on ME broth (Fig E and Fig F in S2 File). Likewise with Pf, Fp repressed production of MnP activity by Jl and Pc on wood-LNAS medium. Three-species co-cultures, instead, presented different effects on MnP production patterns depending on the white rot fungal combinations (Fig E and Fig F in S2 File). Combinations of FpPrTa, FpPrPc, FpPrJl and FpPrPf showed cyclic production of MnP activity on wood-LNAS, with FpPrTa combination producing the highest activities comparable to MnP activity production by Pr alone (Fig 3A). On ME broth, however, these combinations resulted with lower MnP activities and a delayed cyclic MnP production by Pr (Fig 3B). No MnP activity, however, was observed with three species combinations on ME broth including either Jl or Pf (Fig F in S2 File).

### Enzyme activities: β-glucosidase

**Fig 4:**
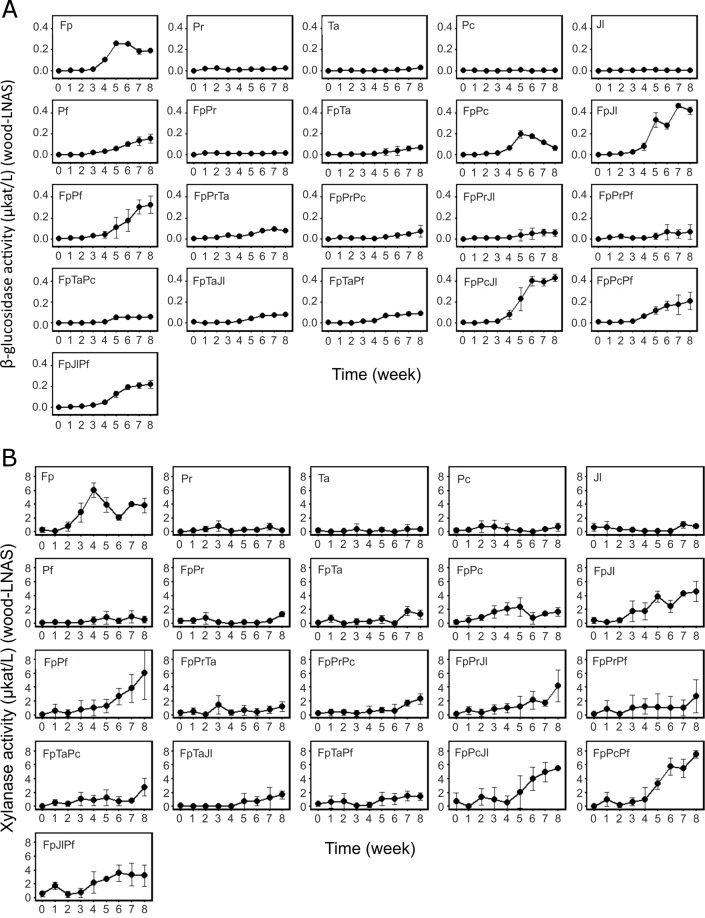
**β-glucosidase activities (A) and endo-β-1,4-xylanase activities (B) in single-species and co-cultures during eight weeks on ground wood-supplemented LNAS medium.** Species abbreviations and combinations, see Table 1 and Table 2. Mean values of three replicate cultures are presented with standard deviation (error bars).

In single-species cultures, Fp and Pf produced detectable activities of β-glucosidase on ground wood-LNAS medium (Fig 4A). No detectable β-glucosidase activity was, however, observed in single-species cultures of the white rot species Pr, Ta, Pc, or Jl. In the two-species and three-species co-culture combinations with Fp including Pr or Ta or both, presence of the white rot species apparently caused a negative effect on β-glucosidase activity, particularly after the fourth week of cultivation (statistically significant difference, *P*<0.05; Fig 4A). On the contrary, presence of Jl or Pf apparently enhanced β-glucosidase activity production by Fp, whereas Pc had no effect on this enzyme activity (Fig 4A, Table B and Fig G in S2 File). Two-species combination of FpPc demonstrated similar activities as Fp alone, thus indicating no clear effect by the presence of Pc on total β-glucosidase activity. The three-species combination of FpJlPf followed similar β-glucosidase activity pattern as observed with FpPf, thus indicating strong influence by the combination of FpPf on β-glucosidase activity levels. Overall, production of β-glucosidase upon interactions of *F*. *pinicola* and the white rot species on wood-LNAS showed variations depending on the fungal combinations used.

### Enzyme activities: Xylanase

In single-species cultures, Fp produced high xylanase activities on wood-LNAS with the increase starting from the second week of cultivation (Fig 4B). The white rot species, on the contrary, demonstrated little to no detectable xylanase activities (Fig 4B, Table B and Fig G in S2 File) whereas in their two and three-species combinations, the white rot species may have affected the observable xylanase activity. In co-cultures including Pr or Ta or both, xylanase activities followed a similar low activity production pattern as was observed for white rot single-species cultures (Fig 4B, Fig G in S2 File). In accordance to the pattern of β-glucosidase production in co-cultures on wood-LNAS medium, presence of the white rot species Jl, Pf and Pc apparently influenced a delay in the xylanase activity. After five weeks, however, a positive effect was observed (Fig G in S2 File). The number of species in co-cultures was irrelevant to the pattern of xylanase activities observed during the cultivation period (eight weeks). The highest values of xylanase activity produced on wood-LNAS were detected at the end of cultivation (after eight weeks) in all co-cultures (Fig G in S2 File).

### Enzyme activities: Effect of coniferous wood as substrate

**Fig 5:**
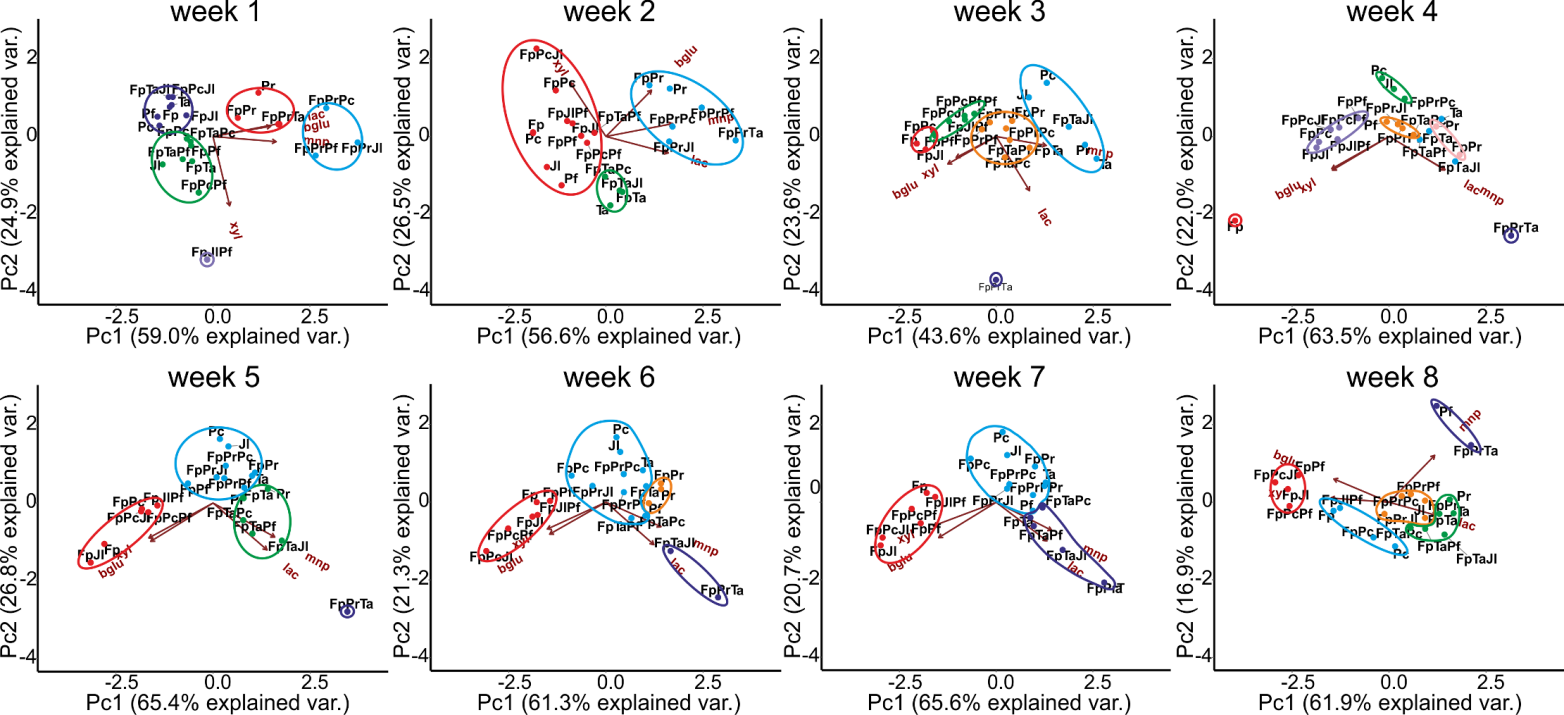
Principal component analysis including clustering of the CAZyme and oxidoreductase activities (xylanase, xyl; β-glucosidase, bglu; laccase, lac; manganese peroxidase, mnp) of single-species and co-cultures produced at each time point on ground wood-supplemented LNAS medium during eight weeks of cultivation. Fungal abbreviations and combinations, see Table 1 and Table 2.

In order to find out potential similarities in enzyme production patterns of the fungal co-cultures, multivariate clustering analysis was performed at each time point (each week of cultivation) for activity values obtained in single-species and combinatorial cultures on wood-LNAS medium. According to visualization of the computation by PCA, clustering of particular species combinations was noticed on the basis of enzyme activities, with the oxidoreductases (laccase and MnP) directing species clustering whereas the hydrolytic enzyme activities (β-glucosidase and xylanase) determined the second main direction and clustering pattern (Fig 5). In this analysis, the two first principal components explained together 80% or more of the variation, except on the third week of cultivation. Thus, the number of fungal species present was shown to influence CAZy glycoside hydrolase (GH) activities on hemicellulose and cellulose-derived oligosaccharides (xylanase and β-glucosidase activities), after two to three weeks of fungal growth. Regarding oxidoreductase activities (laccase and manganese peroxidase), the number of fungal species present in co-cultures was statistically insignificant, whereas the selection of species in the combination affected these activities.

Enzyme activity dynamics was observed as changes in clustering of the fungal combinations over time, while co-cultures including *P*. *radiata* and *T*. *abietinum* together with *F*. *pinicola* (FpPrTa) and the two-species cultivation of *T*. *abietinum* with *F*. *pinicola* (FpTa) were forming a distinct cluster determined by the oxidoreductase activities during weeks 4–7 (purple circle, Fig 5). On week eight, MnP activity determined grouping of the three-species combination FpPrTa with Pf single-species cultures. On the other hand, Fp in combination with Jl, Pf and Pc formed a distinct cluster according to the hydrolytic enzyme activities (red circle, Fig 5), particularly starting from cultivation week five. On week eight, two more enzyme activity groups formed, based on their similarities in laccase activity level (orange and green circles, Fig 5).

Thus, dynamic changes of enzyme activity profiles occurred in the fungal co-cultures, which can be especially observed in the changing positioning, cluster size, and number of fungal combinations grouping in the most variable cluster (blue circle, Fig 5). Three-species co-culture combination including Fp with both of the two strongest oxidoreductase producers, Pr and Ta, was distinct from the other clusters during weeks 3–5 (purple circle, Fig 5), apparently due to strong laccase and MnP activity production by the latter species (Fig C and Fig E in S2 File). Notable was that co-cultures with Fp, as well as Fp single-species cultures, were strong in xylanase and β-glucosidase activities, thereby directed by these activities into a distinct group (in the left side, Fig 5) except in combination with Pc (FpPc).

### End-point pH and production of oxalic acid

On wood-LNAS medium, the culture fluid pH increased in the white rot single-species cultures during the eight weeks of cultivation, initiating from the originally buffered (by sodium succinate) pH 4.5 value to pH values over 6.0 (up to pH 6.2 with Pf), whereas with Fp single-culture and in the two-species combination FpPc, the wood-based cultures were acidified to around pH 3.2 (Fig B in S2 File). Of the white rot species studied, Pc single culture was exceptional while causing no effect on the initial pH of the wood-LNAS medium.

On the more rich malt-extract (ME) medium, however, a more general acidification occurred in co-cultures with Fp, with final pH values decreasing from the initial (pH 5.5) even as low as to pH 1.7, similar to the values obtained in Fp single-species cultures (Table A in S2 File). With white rot single-species cultures, on the contrary, the end-pH values on ME medium were stable (close to initial pH 5.5). Only exception was Pr with lower end-pH values in single-species cultures (pH 4.3). With Pr present in the combinatorial cultures, acidity increased but more moderately than in Fp single-cultures, resulting with end-pH values again close to pH 4 (3.9−4.3). Thus, presence of Pr in the co-cultures could apparently prevent the acidification seen when Fp was cultured alone.

In accordance to the very low pH values observed in Fp single-cultures, the fungus produced large quantities of oxalic acid on the richer ME medium (between 30–62 mM, on week eight; Table A in S2 File). In co-cultures with Pr present, however, accumulated oxalic acid concentrations were not more than 1.9 mM (combination FpPrJl), which is similar to the concentrations (1.0 mM) obtained from Pr single-species cultures. Considering the results, Pr was apparently the only white rot fungus producing detectable amounts of oxalic acid under these conditions.

**Fig 6:**
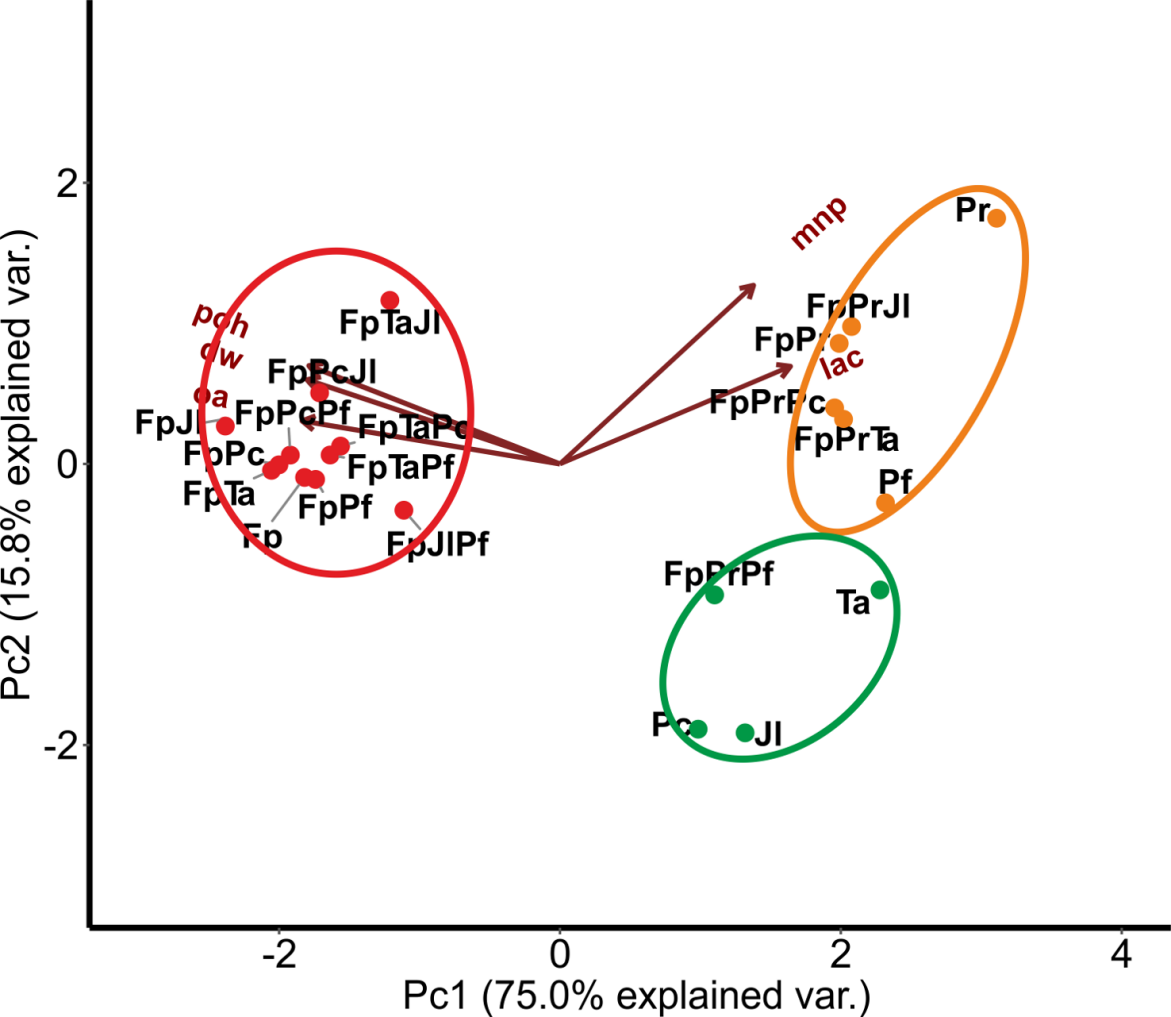
Principal component analysis of acidity (poh), concentration of oxalic acid (oa), production of mycelial mass (dry weight, dw), laccase activity (lac) and manganese peroxidase activity (mnp) determined from co-cultures after eight weeks of cultivation on malt extract liquid medium. Distinct clusters are marked: red circle, Fp single and co-cultures without Pr; orange circle, single-species cultures of Pr and Pf together with co-cultures including Pr—except combination FpPrPf; green circle, white-rot single-species cultures of Ta, Pc, Pf, Jl, and combination FpPrPf. Fungal abbreviations and combinations, see Table 1 and Table 2.

In order to study the relationship of oxalic acid production with culture acidity, mycelial growth and enzyme activities, and regarding fungal combinations in co-cultures, a second clustering analysis was performed after eight weeks of growth on ME medium for the end-point situation (Fig 6). As a result, noticeable clustering effects were observed. Single-species cultures of Fp and combinations with the white rot species, excluding Pr, grouped together forming a distinctive large cluster directed by end-pH, accumulation of oxalic acid and production mycelium biomass (red circle, Fig 6). However, co-cultures including Pr apparently clustered together with Pr single-species cultures, determined by the oxidoreductase activities and forming a distinctive Pr-cluster (orange circle, Fig 6), except for the combination FpPrPf. The latter combination grouped together with single cultures of Ta, Pc and Jl (green circle, Fig 6). The determining factors for fungal culture grouping were the oxidoreductase activities produced by Pr, oxalic acid production by Fp, and production of mycelia (determined as dry weight) in single species and co-cultures.

## Discussion

In this study, interactions of the brown rot fungus *Fomitopsis pinicola* were investigated in co-culture combinations with five white rot fungal species of Agaricomycetes (*Phlebia radiata*, *Trichaptum abietinum*, *Junghuhnia luteoalba*, *Phellinus ferrugineovelutinus* (formerly *P*. *ferrugineofuscus*) and *Porodaedalea laricis* (formerly *Phellinus chrysoloma*), either in two- or three-species combinations. Our intention was to record hyphal interactions and also investigate, how mycelial interactions affect production of wood-decaying enzyme activities in submerged and ground-wood supplemented co-cultures on different carbon sources. Quite opposite to the working hypothesis, our results indicate that in co-cultivations of two to three species interacting, the major impact on enzyme activity profiles was noticed to be influenced by two white rot species, namely *P*. *radiata* and *T*. *abietinum*, while the brown rot species *F*. *pinicola* had less or no impact on their enzyme production. Secondly, it was noticed that on coniferous wood substrate, significant variations were observed weekly in lignocellulose-degrading enzyme activity profiles depending on co-culture age and the species combination.

### Mycelial interactions and wood-decay enzymes

In regard to mycelial growth patterns on agar media, the isolate of *F*. *pinicola* proved to be a supreme colonizer, particularly on malt-extract agar, with hyphae easily extending over the mycelia of the other species studied. Upon these interactions, no visible mycelial blocks were generated against *F*. *pinicola* by any of the white rot species studied. This is somewhat surprising, since it has been noted that saprotrophic wood-decaying fungi readily form mycelial blocks upon hyphal confrontations [6]. However, in our study, the white rot Agaricomycetes species reacted more antagonistically towards each other. Orange-yellow coloured mycelial blocks were generated by *P*. *radiata* against a few of the other white rot species, but not in all cases. Similar species-dependent variations in pigmentation of hyphal fronts in the interaction strategies have been observed with other white rot fungal species [52].

It may be assumed that both the quality (which fungal species and isolates are selected for the combinatorial cultures) and quantity (number of species and isolates in the co-cultures) of fungal combinations would affect production and total activities of the fungal secreted wood-decay enzymes and metabolites. Functional changes and wood-decay processes, however, are difficult to follow *in situ*, in decaying wood in forest sites. The recent long-term surveys in boreal and temperate forests in central and northern Europe, and in Fenno-Scandia, on wood-decay fungal communities and their effects on wood structural and chemical parameters have addressed the dynamics of fungal decomposition of wood [4, 26–28]. Also, pre-inoculated Norway spruce trunks have been subjected as targets for fungal colonization in the long-term forest site studies [2, 26], and trials for analysis of fungal biomass and enzyme activities during the early phase of natural decomposition have been carried out [28–29]. Taking this into account, we designed fungal interactive co-cultures with species noticed in the above mentioned studies to occupy Norway spruce wood as their natural substrate, and either co-existing or dominating this habitat (like *F*. *pinicola*) in boreal forests [2, 4, 27].

### Laccase activities in co-cultures

Previously, activities of extracellular wood-decay oxidoreductases, such as laccase, have been observed to change upon interspecies level fungal interactions on liquid media [52–54]. With the white rot Polyporales species *Trametes versicolor*, it was reported that in co-cultures with other fungal species, laccase activity may be increased at the mycelial interaction zones in comparison to self-paired mycelia on agar plates [54]. The authors stated that increase in laccase activity was more likely a response to the combative situation between species [54]. In our study, the highest laccase activities were observed in co-cultures including two white rot fungal species (*P*. *radiata* and *T*. *abietinum*), and laccase activities were also increasing in some combinations including three species. The increase in laccase activities (or laccase production) upon these interactions could be explained by competition for nutritional and habitat resources, or more simply, as a defence reaction following mycelial confrontations [54–55].

As can be expected, increased laccase activities have been observed in interactive fungal co-cultures of two white rot fungi that also demonstrate strong laccase production alone, in single cultures [53]. This is in accordance to our results obtained with the laccase-producing white rot species *P*. *radiata* and *T*. *abietinum*, and their summative laccase activity effect observed in three-species co-cultures including Fp. It has been noticed that Basidiomycota fungi which are incapable of laccase production in single cultures, may remain incapable in the presence of other species [53]. Accordingly in our study, *F*. *pinicola* as well as Pc (*Porodaedalea laricis*) demonstrated no laccase activity either in single-species cultures or in their paired two-species co-cultures. In the case of *F*. *pinicola*, this is surprising, since three putative laccase (of the five multicopper oxidase) encoding genes are present in the genome of this brown-rot species [11]. However, and in line with our results, no laccase proteins were found in the secretome of *F*. *pinicola* cultivated on poplar wood-containing medium [56]. In conclusion, it may thereby be stated that laccase expression and enzyme activity are not primarily influenced by contact with other fungi or other fungal hyphae in the same habitat. Instead of being a combative factor and defence response, production of laccase may be primarily a general response in white-rot fungi of Polyporales resulting from mycelial growth on their lignocellulose and carbohydrate-containing substrates, as has been suggested before [10, 12].

Furthermore, laccase was produced together with MnP activities by the white rot species *P*. *radiata* and *T*. *abietinum*. Strong oxidoreductase encoding gene expression and enzyme protein production burst–involving a set of lignin-attacking class-II peroxidases and hydrogen-peroxide producing oxidases—has been demonstrated to occur in *P*. *radiata* when growing in solid-state cultures on spruce wood [14]. Thus, it may be suggested that in liquid co-cultures supplemented with ground spruce wood and including either *P*. *radiata* or *T*. *abietinum* or both species, elevated levels of laccase and class-II peroxidase activities indicate on-going strong oxidative, early-phase degradation processes on lignocellulose components and polysaccharides, initiated by the white rot fungal secreted oxidoreductases. The multivariate analyses, as depicted by PCA, accordingly indicated the determinant effect of laccase and MnP production–mainly by *P*. *radiata* and *T*. *abietinum*—as the main directing factors for phenotype grouping of the fungal combinations.

The brown rot Polyporales species *F*. *pinicola* was present in all co-culture combinations, which may explain prevention of laccase activity and enzyme production by a few of the white rot species, except for *P*. *radiata* and *T*. *abietinum*. Surprisingly, no laccase activities were detected for *F*. *pinicola* alone (in single-species cultures) on any of the culture media tested, although laccase-like oxidoreductase (ABTS oxidation) activity and protein expression was reported for another isolate of the species [57], and three laccase encoding genes have been predicted from *F*. *pinicola* genome sequence [11]. Our results of non-detectable laccase activity, however, are supported by a previous study in which laccase activity was not observed in cultures of brown rot fungi cultivated on liquid media [29]. This implies that *F*. *pinicola* laccase production may be repressed under cultivation conditions adopted in our study.

### Production of oxalic acid

Apparently, our results indicate that fungal secreted oxalic acid had an impact not only on the culture medium acidity but also on generation of mycelial biomass and hyphal growth. *F*. *pinicola* was the highest producer of oxalate (30–61 mM concentrations) as quantified from malt-extract medium cultures after eight weeks of growth, whereas minor concentrations were produced by the white rot species *P*. *radiata* (1 mM). Strong production of oxalic acid is a more general ability of brown rot fungi than in white rot species [58–60], and corresponding (over 10 mM) concentrations of oxalate have been reported to accumulate in cultures of other brown rot species [61–62].

In co-cultures on malt-extract liquid medium, *P*. *radiata* prevented acidification to some extent, which may be due to active decomposition of oxalate produced by *F*. *pinicola*. *P*. *radiata* genome includes several oxalate decarboxylase (ODC) encoding genes [14]. Fungal ODC and oxalate oxidase enzymes degrade oxalate to carbon dioxide, and thus may aid white rot fungi to maintain more stable concentrations of oxalic acid and acidity in their environment [60, 63]. No noticeable concentrations of formate (formic acid), which is a degradation product of oxalate, were detected in the co-cultures as an indication of ODC activity. However, ODC activity may not be ruled out completely since formate may have been further degraded to carbon dioxide by fungal formate dehydrogenase (FDH) [64].

### Interactions between brown rot and white rot fungi

Differentiation of the wood-decaying Agaricomycetes to either brown rot or white rot fungi is not so simplified and divided as was previously believed [15, 24], which may explain why the white rot species, *P*. *radiata* and *T*. *abietinum*, formed their own cluster with Fp, which was driven by similarities in activity levels of laccase and MnP, in the coniferous-wood supplemented co-cultures while the other white rot species clustered together with *F*. *pinicola* single-species cultures, with respect to end-point acidity and production of oxalic acid and mycelial mass. On the other hand, some white rot species may be dependent on other fungi for colonization and degradation of their wood substrate environments [2, 4–5, 26]. In the case of the early-colonizing *Porodaedalea laricis* isolate (formerly *Phellinus chrysoloma*) [38] single-species cultures demonstrated very low CAZyme activities which may indicate that in nature, this fungus may live together with other fungal species, in order to obtain nutrients and new hyphal habitats. Presence of other fungal hyphae and their metabolites, or wood degradation products, may thereby stimulate expression of wood-decaying enzymes and promote degradation processes of the “later” coming species [2, 4–5, 27]. In this study, the early-colonizing characterized species *F*. *pinicola* and middle-colonizing species *Phellinus ferrugineovelutinus*, and late-colonizing species *Junghuhnia luteoalba* (see also Table 1) demonstrated higher β-glucosidase activities in two species co-cultures compared to their single-species cultivations.

In regard to the superior colonizing ability of the brown rot species *F*. *pinicola* in our study, it was surprising to find that white rot species (*P*. *radiata* and *T*. *abietinum* in particular) were very capable for mycelial cohabitation and hyphal colonization of the same growth environment (agar plates or liquid media flask cultures) with *F*. *pinicola* without aggressive or strong combative reactions. On the contrary, in our experiments, these three Polyporales species shared more of a mutualistic than combative growth pattern. It has been noticed in long-term (6 to 12 years) surveys that in the boreal forest site of Norway spruce logs pre-inoculated with *F*. *pinicola*, the decaying logs were further occupied by various white rot fungal species–including e.g. *T*. *abietinum* and *Junghuhnia separabilima*–which are more tolerant and competitive for the long-term final degradation of wood [2, 26]. Accordingly in our study, the isolate of *J*. *luteoalba* (initially isolated from decaying Norway spruce wood) was able to coexist together with *F*. *pinicola*, as well as with the more robust white rot fungal species *P*. *radiata* and *T*. *abietinum*.

## Conclusions

In this study, hyphal interactions on solid agar media demonstrated that *F*. *pinicola* mycelium quickly dominates the white rot species in all combinations, regardless of the number of fungal species introduced. Although *F*. *pinicola* hyphae extended in dense white mycelial zones over the hyphae of the white rot fungal species, but simultaneously allowing expansion of the other species’ mycelia, which indicated mutualistic instead of aggressively combating relationships between the fungi studied. The mutualistic or cohabitation strategy was also supported by the fungal enzyme production profiles in co-cultures on the ground-wood supplemented medium, wherein the white rot species *P*. *radiata* and *T*. *abietinum* at first established oxidoreductase activities (laccase and manganese peroxidase), apparently unaffected by the presence of the brown rot species *F*. *pinicola*. On the other hand, a few white rot fungi (*P*. *ferrugineovelutinus* and *J*. *luteoalba*) caused a slight positive effect on the later production of xylanase and β-glucosidase activities in combination with *F*. *pinicola*. These results support that also upon naturally occurring interactions of wood-decay fungi, the rapid oxidative first degradation phase of wood is followed by a later, carbohydrate-active enzyme dominated second decay phase.

Our results imply that interspecies interactions of Agaricomycetes are dynamic factors to be taken into account when unveiling the fungal and enzymatic decay of wood, and in investigating the role of fungi in recycling of lignocellulosic carbon in the forest ecosystems. Our results also indicate that saprotrophic fungi affect each other in multiple ways in their habitats, thus indicating that the mycelial encounters including several species may in fact be beneficial and even promotive for biodegradation of wood and cycling of forest organic carbon.

## Supporting information

S1 File Molecular systematics of the fungal isolates. (DOC)

S2 File Supplementary information and data. (DOC)
