# Supplementary material for: Interactions affect hyphal growth and enzyme profiles in combinations of coniferous wood-decaying fungi of Agaricomycetes
Source: PLoS One. 2017 Sep 27;12(9):e0185171. doi: 10.1371/journal.pone.0185171 (PMC5617175; doi:10.1371/journal.pone.0185171)
Supplement: S1 File — (DOC) [file pone.0185171.s001.doc]

**S1. Molecular systematics of the fungal isolates**

**Methods**

ITS1-5.8S-ITS2 PCR systematics and phylogenetic analysis was performed for the fungal isolates studied (Table 1), in order to verify their identity and species-level taxonomy. Phire Plant Direct PCR kit (Thermo Scientific) method was used for pieces of hyphae (derived from MEA cultures) according to the instructions of the manufacturer. For two isolates (Pr 0043 and Pf 0945), mycelium breaking-DNA extraction method [39-40] was adopted. ITS1 and ITS4 primers (White *et al.* 1990) were used under optimized Phire PCR conditions (Thermo Scientific). Sequencing of both DNA-strands of the PCR products was performed by a service laboratory (DNA Sequencing and Genomics, Institute of Biotechnology, University of Helsinki). ITS sequences were analyzed with BioEdit software version 7.0.5.3 (Tom Hall, Ibis Biosciences). Reference sequences were obtained from NCBI database using BLAST searches (blastn algorithm). Phylogenetic analyses were run in MEGA 6 package (Tamura *et al.* 2013) using ClustalW alignment followed by minimum evolution computing and neighbor-joining with bootstrapping repeat testing for the branching.

**Results**

Obtained ITS1+5.8S+ITS2 sequences are found at ENA (EMBL-EBI) under following accessions: [LT844580] *Fomitopsis pinicola* FBCC1181, [LT844581] *Phlebia radiata* FBCC0043, [LT844582] *Trichaptum abietinum* FBCC0110, [LT844583] *Junghuhnia luteoalba* FBCC1472, [LT844584] *Porodaedalea laricis* FBCC0768, and [LT844585] *Phellinus ferrugineovelutinus* FBCC0945. According to ITS-region sequence similarity and phylogeny, the isolate FBCC0768 originally identified to the species *Phellinus chrysoloma*, however, represented higher ITS-sequence identity to isolates of the genus *Porodaedalea*. At species level, the most identical taxon was *Porodaedalea laricis* (Fig A in S1 File), thus supporting revision of the species name. The isolate FBCC0945 previously identified to the species *Phellinus ferrugineofuscus* was accordingly re-named at species level to *Phellinus ferrugineovelutinus*, due to ITS sequence similarity pointing to the latter species (Fig A in S1 File). This is not astonishing, since molecular systematic studies have indicated that fungal isolates addressed to *Phellinus* genus/genera group are under taxonomic re-organization according to their ITS sequences (Tomšovský *et al.* 2010). Except for these two isolates of *Phellinus* and *Porodaedalea*, the other fungi of this study retained their taxa and species-level identities (Fig A in S1 File).

**Fig. A** Phylogenetic tree of the ITS-sequences of the Agaricomycetes fungal isolates and species studied. Sequenced ITS1-5.8S-ITS2 rDNAs (FBCC isolate numbers) were positioned with their nearest identical reference taxon sequences (NCBI GeneBank accessions). Bootstrap values (100 replications) are marked for the nodes. ITS-sequence of *Ustilago maydis* (Ustilaginomycetes) was used as outgroup. Scale bar represents 0.05 nucleotide substitutions per position.

**S1. References**

Tamura K, Stecher G, Peterson D, Filipski A, Kumar S. MEGA6: Molecular Evolutionary Genetics Analysis version 6.0. Mol Biol Evol 2013;30:2725-9.

Tomšovský M, Sedlák P, Jankovský L. Species recognition and phylogenetic relationships of European *Porodaedalea* (Basidiomycota, Hymenochaetales). Mycol Prog. 2010;9:225-33.

White TJ, Bruns T, Lee S, et al. Amplification and direct sequencing of fungal ribosomal RNA genes for phylogenetics. In: Innis MA, Gelfand DH, Sninsky JJ, et al. (eds.) PCR Protocols: A Guide to Methods and Applications*.* 1990; pp.315−22. New York: Academic Press, Inc.
