# Supplementary material for: Interactions affect hyphal growth and enzyme profiles in combinations of coniferous wood-decaying fungi of Agaricomycetes
Source: PLoS One. 2017 Sep 27;12(9):e0185171. doi: 10.1371/journal.pone.0185171 (PMC5617175; doi:10.1371/journal.pone.0185171)
Supplement: S2 File — (DOC) [file pone.0185171.s002.doc]

**S2. Supplementary file 2, information and data**

**Table A** End-point pH, mycelial dry weight and production of oxalic acid in single-species and co-cultures on malt extract medium determined on cultivation week eight. Values represent the mean and standard deviation (STD) of three parallel cultures. Species abbreviations are depicted in Table 1. ME medium, unincolulated medium; nd, not determined; 0, below detection limit.

| **Fungal combination** | **Mycelial dry weight** | | **pH** | | **Oxalic acid** | |
| --- | --- | --- | --- | --- | --- | --- |
|  | **(mg)** | **STD** | **value** | **STD** | **(mM)** | **STD** |
| Fp | 112.6 | 0.013 | 1.60 | 0.022 | 58.0 | 3.125 |
| Pr | 60.4 | 0.010 | 4.26 | 0.053 | 1.1 | 0.148 |
| Ta | 39.4 | 0.004 | 5.50 | 0.012 | 0 | nd |
| Pc | 51.2 | 0.003 | 5.10 | 0.017 | 0 | nd |
| Jl | 41.2 | 0.011 | 5.49 | 0.052 | 0 | nd |
| Pf | 42.0 | 0.004 | 5.76 | 0.025 | 0 | nd |
| FpPr | 67.3 | 0.008 | 3.89 | 0.054 | 0 | nd |
| FpTa | 124.4 | 0.008 | 1.53 | 0.090 | 61.4 | 10.189 |
| FpPc | 150.8 | 0.002 | 1.62 | 0.024 | 47.7 | 4.975 |
| FpJl | 164.7 | 0.019 | 1.53 | 0.017 | 60.1 | 4.369 |
| FpPf | 152.8 | 0.014 | 1.73 | 0.041 | 35.8 | 2.068 |
| FpPrTa | 53.1 | 0.005 | 4.17 | 0.103 | 0 | nd |
| FpPrPc | 52.0 | 0.005 | 3.96 | 0.078 | 0 | nd |
| FpPrJl | 69.3 | 0.008 | 3.92 | 0.131 | 0.6 | 0.887 |
| FpPrPf | 61.8 | 0.008 | 3.86 | 0.046 | 0.3 | 0.430 |
| FpTaPc | 150.2 | 0.005 | 1.60 | 0.041 | 32.6 | 4.993 |
| FpTaJl | 159.2 | 0.006 | 1.64 | 0.026 | 35.1 | 10.040 |
| FpTaPf | 153.2 | 0.007 | 1.68 | 0.076 | 34.1 | 2.526 |
| FpPcJl | 168.4 | 0.017 | 1.56 | 0.049 | 36.1 | 18.646 |
| FpPcPf | 166.2 | 0.022 | 1.66 | 0.017 | 38.5 | 11.738 |
| FpJlPf | 106.7 | 0.039 | 1.73 | 0.005 | 29.9 | 2.518 |
| ME medium | 36.0 | 0.003 | 5.32 | 0.034 | 0 | nd |

**Table B** The highest lignocellulose-modifying enzyme activities in wood-LNAS, and laccase and MnP activities in 2% malt extract broth (ME) cultures. Values represent the mean value and standard deviation (STD) of three parallel cultures. Species abbreviations are depicted in Table 1.

^1^detection limit was 0.1 µkat/l

^2^calculated theoretical detection limit was 1.67 µkat/l

**Fig. A** Plan for inoculation of the three and two species co-cultures on agar media plates.

**Fig. B** Cultivation end-point pH in single-species and co-cultures on wood-LNAS medium. Species abbreviations are depicted in Table 1. –cont, un-inoculated wood-LNAS medium.


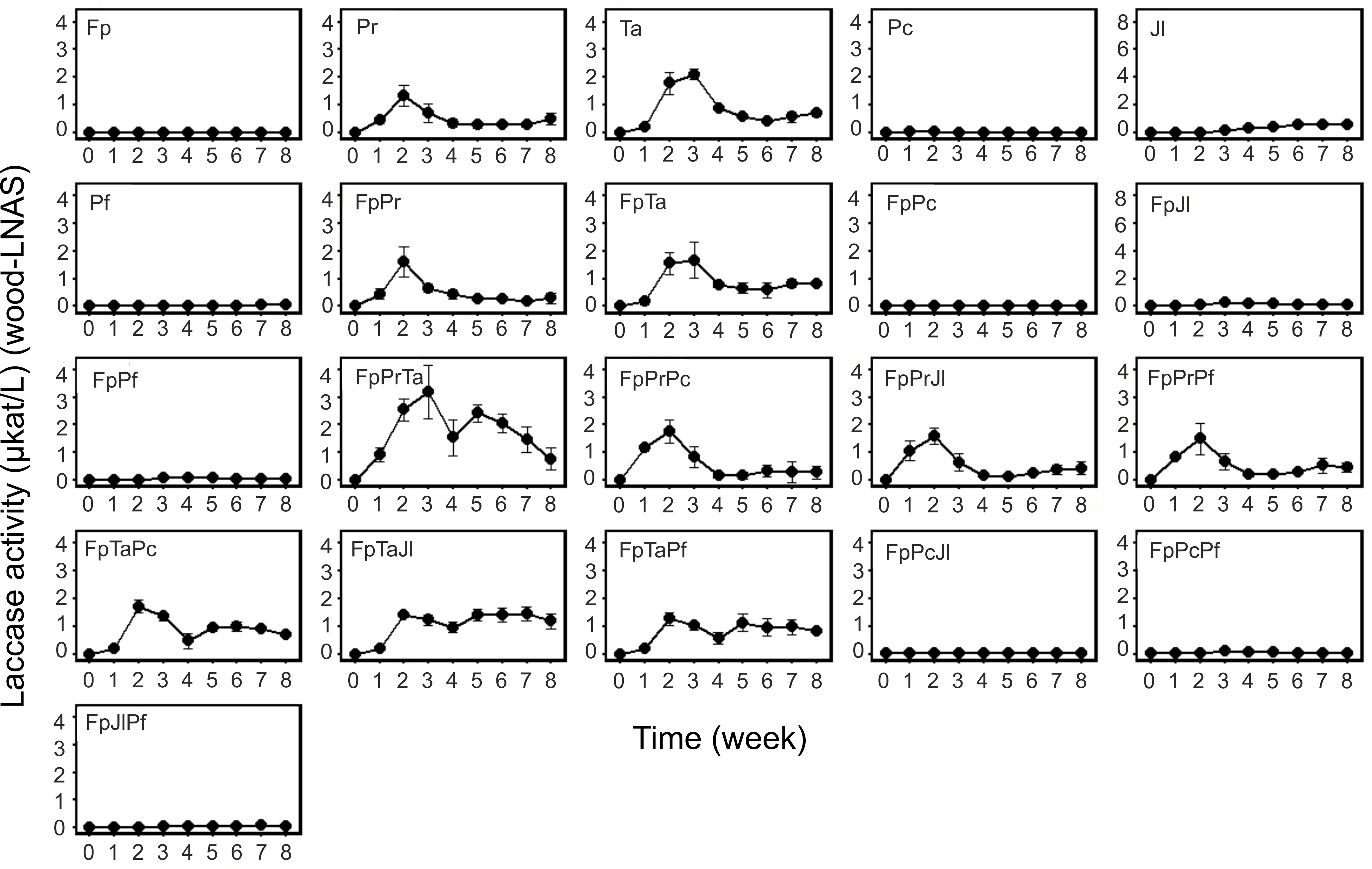


**Fig. C** Laccase activities in single and co-cultures on the semi-solid wood-LNAS medium. Species abbreviations are depicted in Table 1 and Table 2 in the manuscript. Mean values of three cultures, error bars: standard deviation.


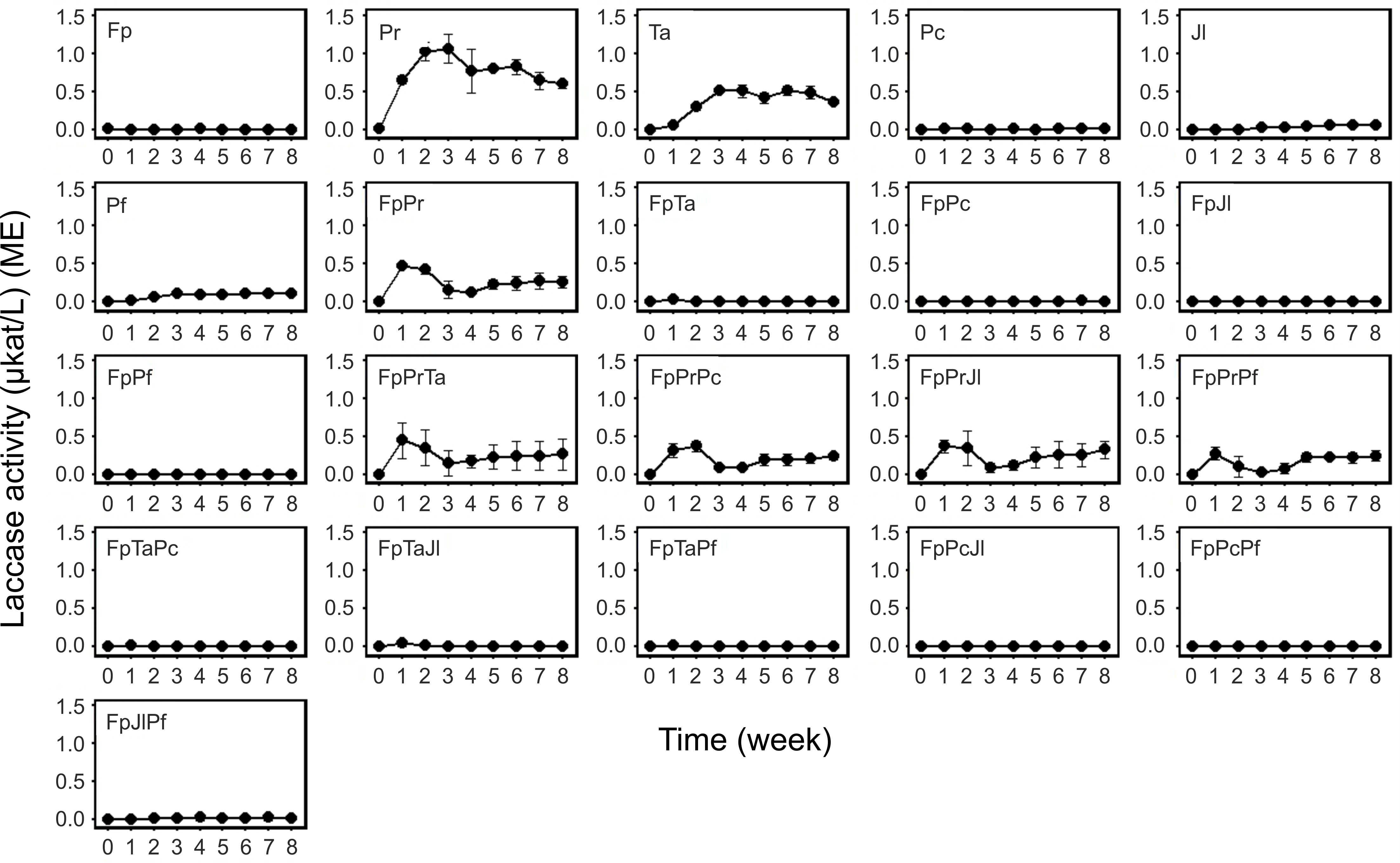


**Fig. D** Laccase activities in single and co-cultures on the liquid malt-extract broth medium.


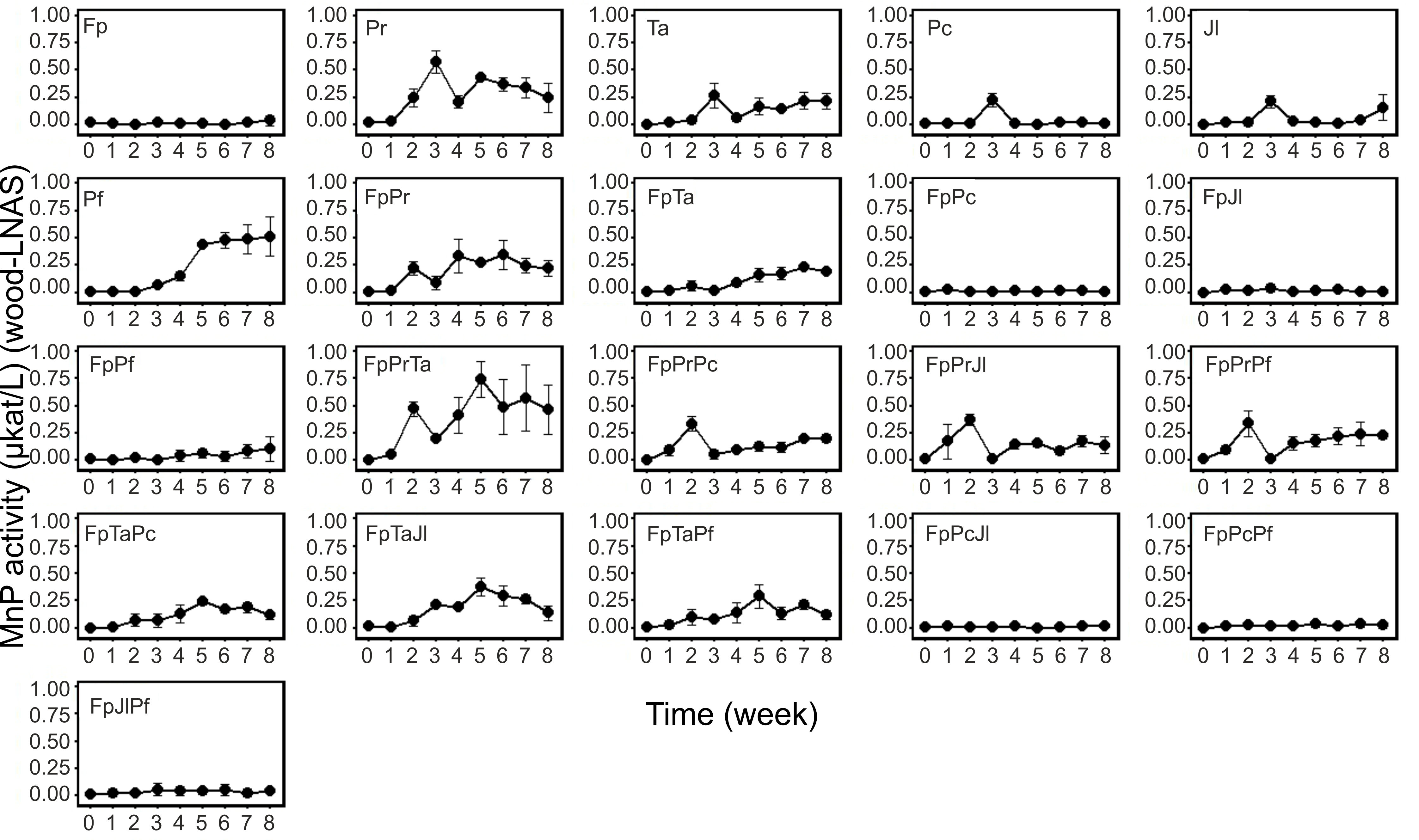


**Fig. E** Manganese peroxidase activities in single and co-cultures on the semi-solid wood-LNAS medium.


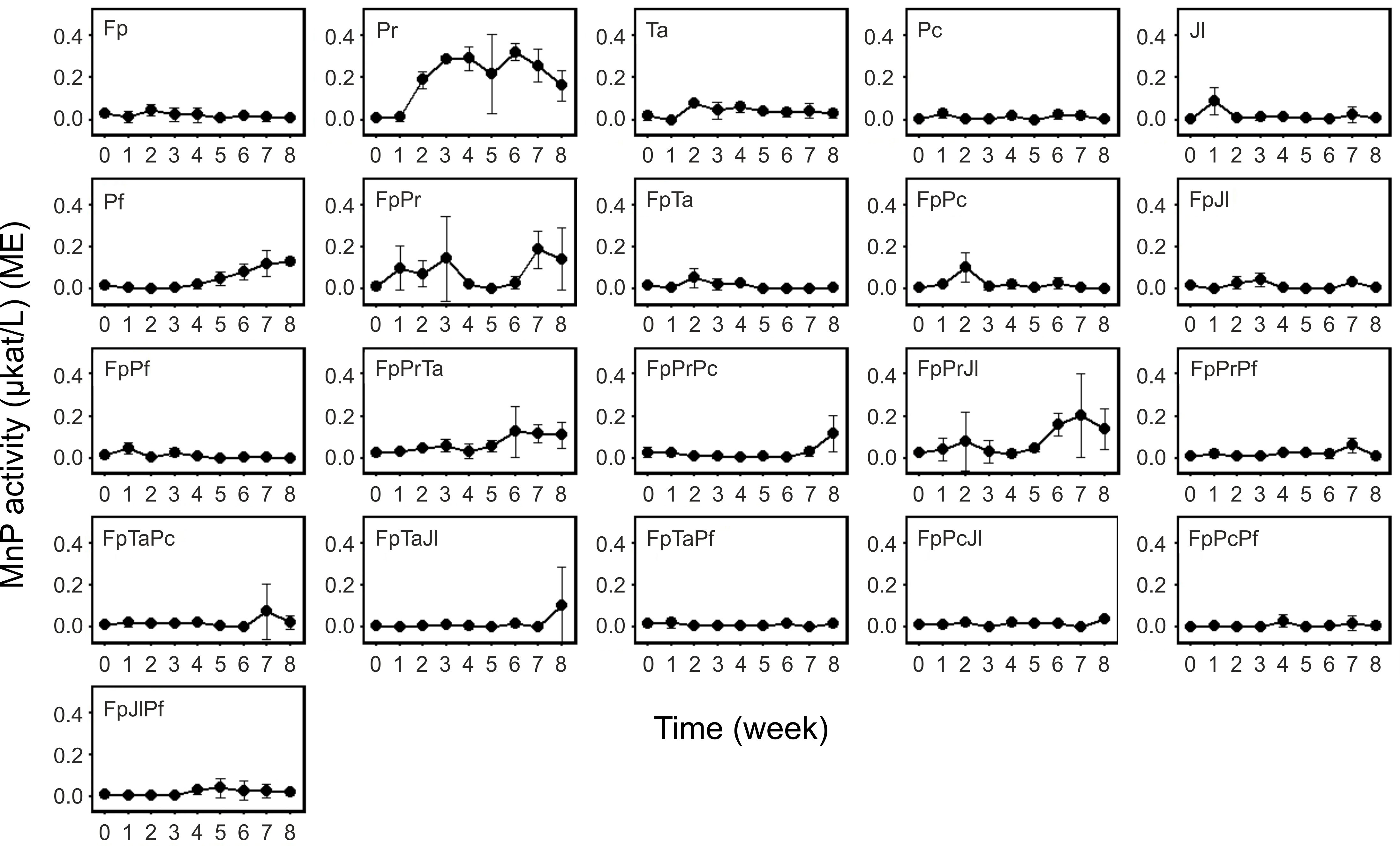


**Fig. F** Manganese peroxidase activities in single and co-cultures on liquid malt-extract medium.


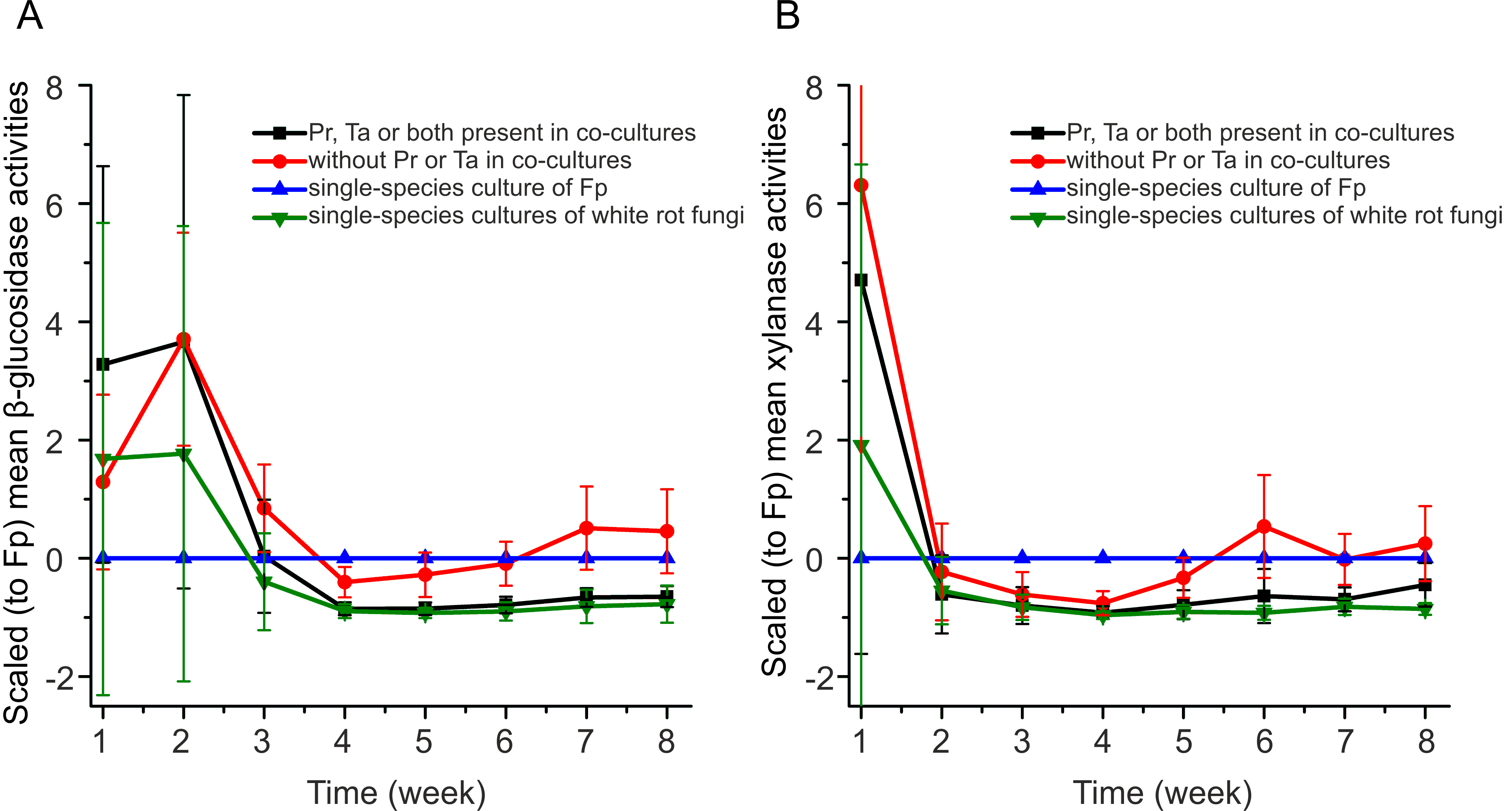


**Fig. G** β-glucosidase (A) and endo-β-1,4-xylanase (B) activities scaled to Fp produced activities, in single-species and co-cultures during eight weeks on ground wood-supplemented LNAS medium. Blue line: Fp activity detected each week as scaled to 0 level, to aid in comparison of the activity pattern changes. Black line: Pr, Ta or both present in the co-cultures. Red line: without Pr or Ta in the co-cultures. Green line: single-species cultures of Pc, Pf or Jl. Fungal abbreviations, see Table 1 and Table 2.


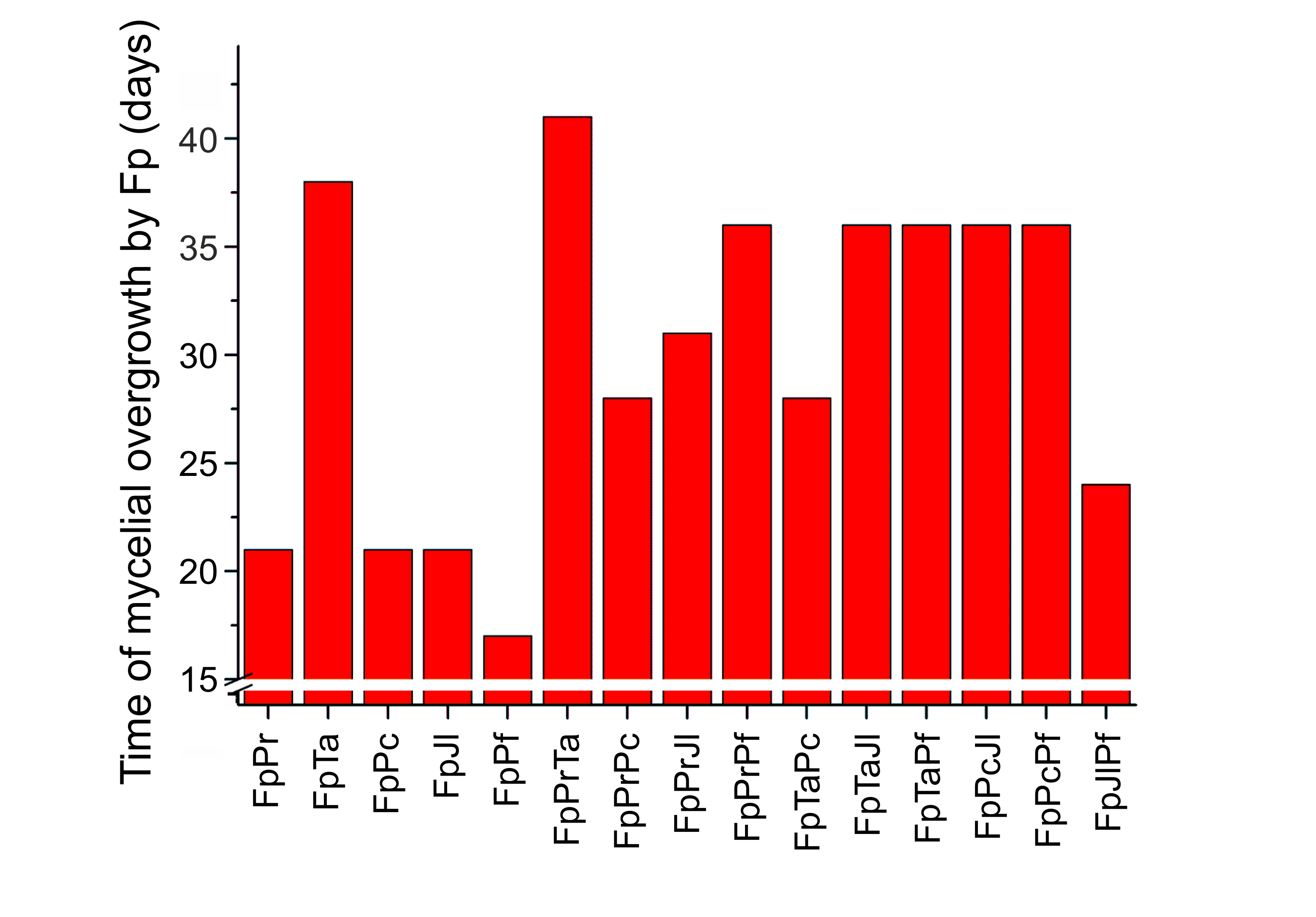


**Fig H** Hyphal extension of *Fomitopsis pinicola* over the white rot fungal species in co-culture combinations on malt-extract agar medium. Fungal abbreviations, see Table 1 and Table 2.
